# Enabling Quechua speech in humanoid social robots through fine-tuned neural TTS on the UBTech Walker Tienkung

**DOI:** 10.3389/frobt.2026.1909076

**Published:** 2026-09-30

**Authors:** Percy Castro-Mejia, William Atalaya-Urrutia, Alejandro Cruzata-Martinez, Erick Salazar-Montoya, Alberto Jimenez-Garcia, Juan Arcila-Diaz

**Affiliations:** Universidad Señor de Sipán, Chiclayo, Peru

**Keywords:** fine-tuning low-resource languages, humanoid social robot, human-robot interaction, indigenous language technology, low-resource speech processing, neural text-to-speech, quechua speech synthesis

## Abstract

Humanoid social robots deployed in multilingual societies—particularly in indigenous communities—face a critical linguistic gap: their speech interfaces are predominantly designed for high-resource languages, excluding hundreds of millions of indigenous language speakers from equitable human-robot interaction. This study presents the first neural text-to-speech (TTS) system for Southern Quechua—the most extensive indigenous language family of the Americas, with more than ten million active speakers—deployed on a humanoid social robot. XTTS-v2, a multilingual neural TTS model, was fine-tuned on 22,727 validated utterances (30.5 h, 77 speakers) of the Cusco-Puno dialect, located in southern Peru, through a tokenizer adaptation that leverages the phonological overlap between Quechua and Spanish. Fine-tuning over 15 epochs on an NVIDIA A100 40 GB GPU achieved a 35.0% reduction in training cross-entropy loss (4.084 → 2.652), confirming effective adaptation to Quechua phonology and morphology with stable training dynamics across 35,000 gradient update steps. Inference latency was evaluated on two hardware environments: the training GPU (mean latency: 2,037 ms; Real-Time Factor (RTF) = 0.472) and the embedded SoC of the UBTech Walker Tienkung humanoid robot (Jetson AGX Orin 64 GB; mean latency: 5,913 ms; RTF = 0.961). Both configurations achieved a mean RTF below 1.0, demonstrating real-time Quechua speech synthesis directly on the robot’s hardware without external computing infrastructure. Intelligibility evaluation using Whisper large-v3 with automatic language detection yielded a Word Error Rate of 147.4% and a Character Error Rate of 69.1%; these values reflect the absence of a native Quechua automatic speech recognition (ASR) model rather than the quality of TTS synthesis. These results establish the first quantitative benchmark for TTS in American indigenous languages on a humanoid robotic platform and provide a reproducible framework—encompassing training configuration, tokenizer adaptation, and deployment pipeline—for extending neural speech synthesis to other low-resource languages of the Americas.

## 1 Introduction

Within the global context, of the approximately 7,000 languages spoken worldwide, fewer than 100 are supported by modern automatic speech processing systems, generating a structural exclusion that affects billions of people who constitute the linguistic majority of the planet (Zevallos et al., 2022; Joshi et al., 2020). This inequality is not coincidental: the development of speech technologies—text-to-speech (TTS) synthesis, automatic speech recognition (ASR), and natural language processing—has historically been concentrated in languages with large digital data collections and consolidated commercial markets, leaving the majority of the world’s linguistic diversity without technological representation. The consequences are concrete: in societies where access to education, healthcare, and public services is increasingly mediated by voice interfaces, the absence of support for indigenous and minority languages constitutes a real barrier to full civic and digital participation (Pine et al., 2025). Humanoid social robots, as emerging platforms for human-robot interaction (HRI) in public and institutional environments, are not exempt from this structural problem: if voice interfaces are not designed to operate in the native languages of the communities they serve, their deployment risks reproducing existing patterns of exclusion (Pine et al., 2025).

Quechua (ISO 639-3: que) is the largest indigenous language family of the Americas, with more than ten million active speakers distributed across Peru, Bolivia, Ecuador, Colombia, and Argentina (Paccotacya-Yanque et al., 2022). Despite this demographic presence, Quechua remains severely underrepresented in speech technology. Quechua operates through a word-construction system based on layers of suffixes chained to a root: a single word can simultaneously encode tense, grammatical person, case relations, evidentiality markers, and discourse relations, producing lexical forms of high phonological and structural complexity (Ortega et al., 2026). Southern Quechua (Qusqu-Qullaw), spoken in the Cusco and Puno regions of Peru, is the most computationally documented dialect and constitutes the focus of the present study. Despite recent advances in the construction of Quechua speech corpora (Paccotacya-Yanque et al., 2022; Zevallos et al., 2022) and initial neural synthesis experiments (Ortega et al., 2026), no prior work has documented the deployment of a Quechua TTS system on any robotic platform.

The present study makes four primary contributions. First, we present the first fine-tuned neural TTS model for Southern Quechua, developed by adapting XTTS-v2 (Casanova et al., 2024) on 22,727 validated utterances (30.5 h) of the Cusco-Puno dialect via a tokenizer patch that redirects Quechua input to the Spanish preprocessor—exploiting the phonological overlap between both languages—executed on an NVIDIA A100 GPU (40 GB HBM2). This adaptation strategy is novel in that it enables effective fine-tuning for a language entirely absent from the base model’s training data without requiring a custom tokenizer or phoneme dictionary. Second, we provide the first quantitative benchmark for inference latency and Real-Time Factor (RTF) for Quechua TTS on both a data center GPU (NVIDIA A100 40 GB; RTF = 0.472) and the embedded SoC of the UBTech Walker Tienkung humanoid robot (Jetson AGX Orin 64 GB; RTF = 0.961), demonstrating real-time synthesis directly on robotic hardware without external computing infrastructure. Third, we document the complete deployment pipeline—encompassing corpus preprocessing, tokenizer adaptation, training stability configuration, and checkpoint loading procedure—as a reproducible framework for extending neural speech synthesis to other low-resource languages of the Americas. Fourth, we document and analyze the research gap at the intersection of TTS for indigenous languages and humanoid robotics, providing the first empirical evidence that general-purpose ASR systems such as Whisper large-v3 produce systematically inflated WER/CER values for unsupported indigenous languages, and establishing methodological guidelines for future evaluation in this domain.

## 2 Background and related work

### 2.1 Speech technology for quechua

Research in automatic Quechua speech processing is both recent and scarce. The Siminchik corpus, described in Paccotacya-Yanque et al. (2022), constitutes the foundational resource of the field: 97.5 h of spontaneous Southern Quechua speech drawn from radio broadcast programs, representing the most extensive aligned speech-text collection available for this language at the time of its publication. Using this corpus, the first ASR systems based on hidden Markov models achieved word error rates ranging from 12.7% to 22.3%, demonstrating both the feasibility of automatic recognition for Quechua and the challenges posed by dialectal variability and data scarcity (Paccotacya-Yanque et al., 2022).

In the domain of ASR, Zevallos et al. (2022) proposed data augmentation strategies specifically designed for low-resource Quechua, achieving significant reductions in Word Error Rate (WER) through speed perturbation, controlled noise addition, and corpus mixing. In the domain of synthesis, Ortega et al. (2026) presented the first systematic study of neural TTS for Southern Quechua, comparing the XTTS-v2 and F5-TTS architectures on the combined Siminchik-Lurin corpus and reporting Mean Opinion Score (MOS) naturalness scores between 3.4 and 3.8 (Ortega et al., 2026). The work of Ortega et al. (2026) constitutes the most direct technical precedent for the present study; the differential contribution of our work lies in the integration of the synthesis model into a humanoid robotic platform with real-time latency evaluation and deployment via ROS 2 — dimensions absent from prior studies.

The Collao emotional speech corpus in Quechua by Paccotacya-Yanque et al. (2022) provides a complementary perspective: although its primary objective is dimensional emotion recognition, the corpus documents acoustic properties of Southern Quechua—including fundamental frequency distribution, energy patterns, and syllable duration—that inform the design of TTS systems for this dialect. The corpus published by Huaman et al. (2026) in the Mozilla Data Collective under a CC0 license comprises 22,727 validated utterances from 77 unique speakers of the Cusco-Puno dialect (code qxp), representing a significant advance in the availability of collaborative data for Southern Quechua. Its greater speaker diversity and acoustic quality, relative to previous versions of the corpus, make it the most suitable resource currently available for fine-tuning neural TTS systems for this dialect.

### 2.2 Speech synthesis for low-resource languages

Neural speech synthesis for languages with scarce computational data resources represents one of the most active fields in contemporary speech processing. Pine et al. (2025) provide the most comprehensive review published to date on this challenge in the context of indigenous languages, identifying four principal technical obstacles: the scarcity of annotated corpora, the phonological complexity of many indigenous languages, the absence of adapted text normalization tools, and evaluation problems arising from the limited availability of native speakers for perceptual studies (Pine et al., 2025). The authors also introduce the EveryVoice TTS toolkit, designed specifically for synthesis in low-resource languages, and document its application to three Canadian indigenous languages with distinct phonological characteristics.

In the domain of neural architectures, the evolution from the autoregressive models of the Tacotron family (Shen et al., 2018) toward non-autoregressive systems such as FastSpeech 2 and VITS (Galdino et al., 2025; Kim et al., 2021) has enabled a reduction in training data requirements through improved capture of prosodic dependencies. Nevertheless, even the most data-efficient models require corpora of tens of hours for languages with no prior representation in the base model, which precludes most indigenous languages from direct deployment. Cross-lingual transfer via fine-tuning of pretrained multilingual TTS models—the central strategy of the present work—has demonstrated viability in low-resource settings for Arabic (Casanova et al., 2024), Swahili, Luganda, and Vietnamese (Pham Ngoc et al., 2023), where fine-tuning base models with 10–50 h of data yields quality comparable to that obtained with monolingual models trained on hundreds of hours.

HiFi-GAN (Kong et al., 2020) establishes the standard in neural vocoders for high-fidelity synthesis. Its multi-period discriminator architecture enables modeling of the periodic patterns of speech with a computational efficiency that makes it particularly suited for fine-tuning with limited data and for inference on embedded hardware. The generalization property of HiFi-GAN to speakers unseen during training—documented in the original work of Kong et al. (2020) — is especially relevant for Quechua, given the speaker diversity present in the available corpora.

### 2.3 Speech synthesis for indigenous languages: state-of-the-art review

The work of Pine et al. (2025) on speech synthesis for indigenous language education represents the most systematic contribution to the field, documenting the development of TTS systems for Canadian languages with fewer than 500 remaining fluent speakers. Their findings are directly relevant to Quechua: the authors demonstrate that perceptual evaluation with native speakers—rather than relying exclusively on automatic metrics—is indispensable for validating TTS systems in languages where the evaluation ASR models are also of low quality. This methodological limitation equally affects the present work and motivates the perceptual evaluation with native speakers planned as future work.

Beyond Quechua, precedents exist for TTS in other indigenous languages of the Americas, although none in a robotic context. Recent studies have addressed Nahuatl, Ecuadorian Kichwa, and several languages of Canada and the United States, employing in all cases fine-tuning strategies on multilingual base models with corpora ranging from 5 to 50 h. The consistency of results across these studies—which report substantial MOS improvements over zero-shot baselines, with values between 3.1 and 3.9 depending on the language and corpus—provides the comparative context for interpreting the results of the present study. Ortega et al. (2026) report naturalness scores for Quechua ranging from 3.4 to 3.8 MOS with XTTS-v2 and F5-TTS respectively, values that serve as a direct reference for our experimental results.

### 2.4 Voice interfaces in humanoid social robots

Voice is the primary communication modality in humanoid social robots. The comprehensive review by Amirova et al. (2021) covering a decade of research with the NAO robot documents the centrality of the speech interface in the most frequent application contexts—education, therapy, and social assistance—and the systematic limitation of NAO’s voice systems to European languages, with scarce exceptions in Japanese and Mandarin Chinese (Amirova et al., 2021). This linguistic concentration reproduces, at the level of robotic interaction, the asymmetries documented in speech technology more broadly, and constitutes a specific barrier to the deployment of social robots in non-Anglophone and non-Europeanized communities.

In the context of South Asian languages, recent work demonstrates the feasibility of conversational TTS systems in humanoid robots for languages other than English. Hegde et al. (2022) implemented a Hindi spoken conversational system on the Mitra humanoid robot, integrating automatic speech recognition, conversational artificial intelligence, and text-to-speech synthesis into a complete pipeline. Similarly, Ullah et al. (2024) developed Disha, a bilingual Bengali-English humanoid virtual assistant oriented toward the banking sector in Bangladesh, explicitly noting that no prior work had adapted AI-powered virtual assistants for Bengali. Both studies share with the present work the motivation of extending robotic speech interfaces beyond English; the fundamental difference lies in the fact that Hindi and Bengali possess substantially greater computational resources than Southern Quechua, and that neither study addresses deployment on embedded hardware in a physically autonomous robot with real-time latency evaluation.

Pourfannan et al. (2023) present one of the few studies published in Frontiers in Robotics and AI that directly address robot speech comprehension in multilingual scenarios. The authors demonstrate that speech time expansion—a reduction in speaking rate—significantly improves comprehension in robots speaking simultaneously in two languages, with effects that interact with the acoustic characteristics of the respective languages (Pourfannan et al., 2023). Although the context differs from the present work, their findings on optimal prosodic parameters for robotic intelligibility inform the design of the synthesis system for Quechua, particularly the adjustment of speech rate in the post-processing module.

Research on optimal response times in conversational HRI establishes that latencies below 300 ms are perceived as natural by human interlocutors, with optimal times close to 200 ms for fluent turn-taking (Skantze, 2021; Aylett and Romeo, 2023). This threshold, derived from studies of spoken dialogue systems involving both humans and robots, defines the central design criterion for the inference pipeline presented in Section 3 and the success criterion for the latency benchmark reported in the results section.

### 2.5 Model optimization for embedded inference

Neural network quantization as a strategy for reducing memory requirements and accelerating inference on resource-constrained hardware has been extensively documented in the machine learning literature. Dynamic 8-bit integer (INT8) quantization via ONNX Runtime—the strategy adopted in the present work—transforms FP32 weights to INT8 prior to inference and computes the scale and zero-point parameters of activations at runtime, without requiring a calibration dataset. The AdamW optimizer with weight decay λ = 10^−2^, adopted in the fine-tuning of XTTS-v2, provides a decoupling of weight decay from the gradient update that improves model generalization in the Quechua domain and is particularly beneficial for fine-tuning large transformers with reduced corpora, where standard L2 regularization tends to degrade performance.

Model export to ONNX format via torch.onnx.export and its subsequent optimization with ONNX Runtime has been shown to reduce transformer model inference latency by factors of between 1.5× and 3× on hardware with SIMD integer instruction support, with quality losses below 2% on natural language processing and speech synthesis tasks (Kim et al., 2021).

### 2.6 Research gap

The preceding review enables precise identification of the gap the present study aims to address. First, no prior work has deployed a TTS system for Quechua—nor for any other indigenous language of the Americas—on a humanoid robotic platform. Second, existing speech synthesis studies for indigenous languages evaluate models under static laboratory conditions, without accounting for the latency constraints imposed by real-time HRI. Third, the multilingual social robotics literature has not addressed the dimension of Andean indigenous languages. The present work operates at the intersection of these three gaps: Quechua speech synthesis, deployment on a humanoid robot, and evaluation under real-time conditions compatible with conversational HRI.

## 3 Method

This section describes the complete set of resources, procedures, and methodological decisions underpinning the present study. The system design follows a sequential pipeline organized into six components, summarized in Figure 1: robotic platform, Quechua speech corpus, model architecture, fine-tuning, export and embedded optimization, and ROS 2 integration. Sections 3.1 through 3.4 correspond to the experimentally validated components of the system, while Section 3.5 document the planned architecture—ONNX export, INT8 quantization, and ROS 2 integration—as immediate future work.

**FIGURE 1:**
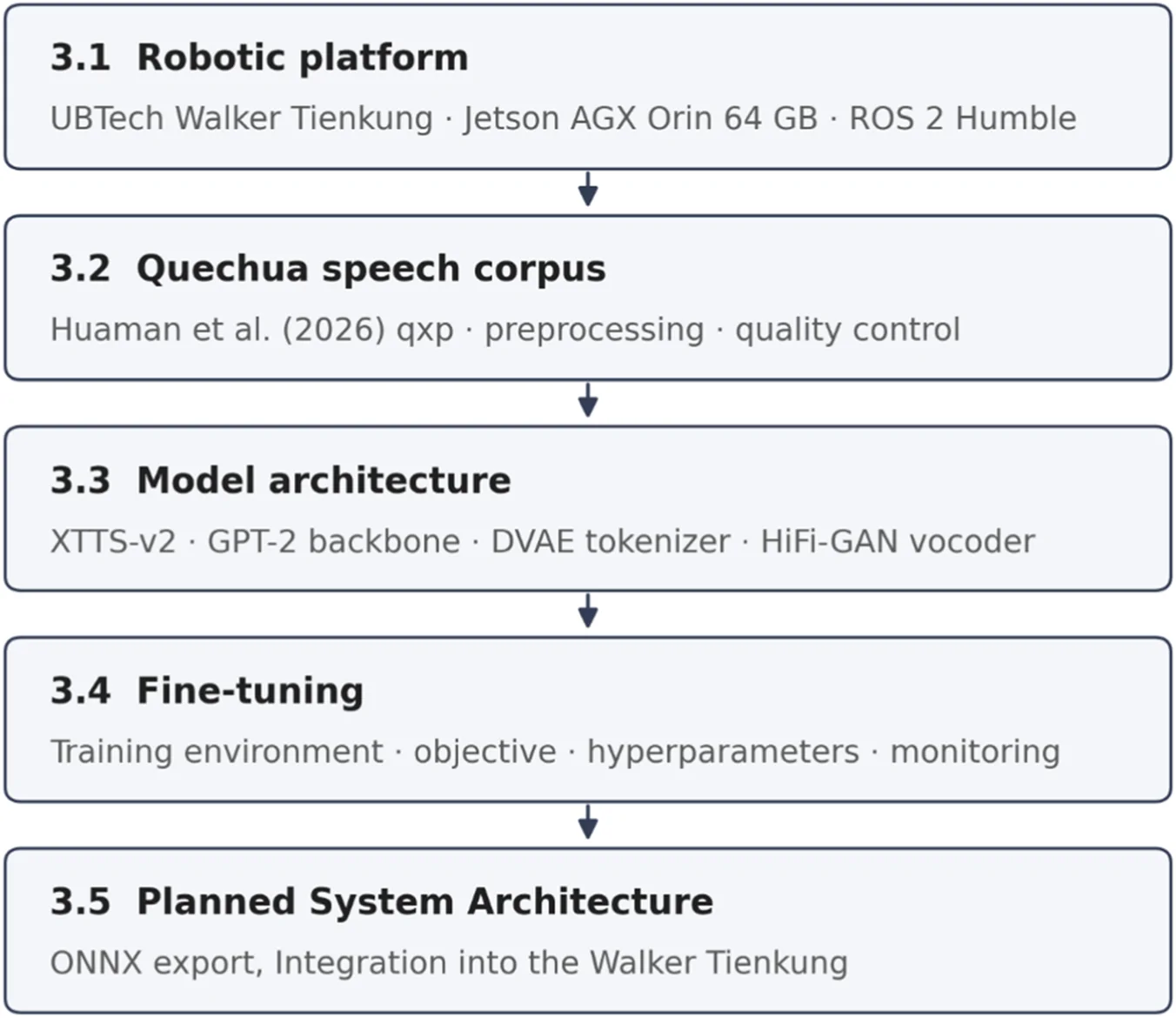
Method architecture overview.

The methodological novelty of this work operates on three levels. At the linguistic level, we introduce a tokenizer redirection strategy that enables fine-tuning of a multilingual TTS model on a language entirely absent from its pretraining data, without requiring a custom phoneme dictionary or orthographic parser. At the hardware level, we provide the first latency characterization of a Quechua TTS model on embedded robotic hardware, establishing RTF as the primary deployment feasibility criterion. At the evaluation level, we document and characterize the systematic failure modes of general-purpose ASR systems when applied to synthesized speech in unsupported indigenous languages, contributing to the methodological discussion on how to evaluate TTS quality in the absence of native ASR support.

### 3.1 Robotic platform

The system was designed and integrated specifically for the UBTech Walker Tienkung humanoid robot (UBTech Robotics, Shenzhen, China). The robot operates on Ubuntu 22.04 LTS and the ROS 2 Humble middleware environment. Its Voice Processing Module (VPM) enables audio input and output through an ALSA-compatible Linux interface, allowing programmatic control from ROS 2 nodes without additional hardware. The Jetson AGX Orin SoC (64 GB LPDDR5, Ampere architecture, ∼275 TOPS INT8) constitutes the target inference processing unit of the TTS system, enabling local model execution without dependence on cloud services—a critical consideration for deployment in Andean communities with limited connectivity (Skantze, 2021). Table 1 presents a detailed overview of the humanoid robot’s specifications.

**TABLE 1:** Specifications of the UBTech Walker Tienkung relevant to the TTS system.

Component	Specification	Relevance to TTS
SoC	Jetson AGX Orin 64 GB	Target inference platform
GPU (Orin)	Ampere (SM 8.x)	FP16 and TensorRT INT8 support
Unified RAM	64 GB RAM	No VRAM constraint for XTTS-v2 (1.87 GB FP32)
CPU	12-core ARM Cortex-A78AE	Parallel audio processing
AI performance	∼275 TOPS (INT8)	Accelerated inference with TensorRT
OS/Middleware	Ubuntu 22.04 + ROS 2 humble	tts_quechua_node
Audio	VPM/ALSA — 22,050 Hz mono	Direct output without additional drivers

### 3.2 Quechua speech corpus

#### 3.2.1 Data source

The training corpus was constructed from the Quechua speech corpus published by Huaman et al. (2026), available under a CC0 license in the Mozilla Data Collective. The Southern Quechua subset (language code: qxp) provides 22,727 validated utterances from 77 unique speakers of the Cusco-Puno dialect. This dataset was selected over the Siminchik and Lurin corpora (Paccotacya-Yanque et al., 2022; Ortega et al., 2026) on account of its unrestricted license, greater speaker diversity, and superior acoustic quality attributable to the more recent recording infrastructure guidelines of the Mozilla platform.

#### 3.2.2 Preprocessing pipeline

The original MP3 files were converted to 16-bit PCM WAV at 22,050 Hz mono using ffmpeg (-ar 22,050 -ac 1 -sample_fmt s16), the sampling rate required by the XTTS-v2 EnCodec tokenizer (Casanova et al., 2024). The following selection filters were applied: transcript length between 3 and 200 characters (MIN_CHARS = 3, MAX_CHARS = 200), and a minimum of one positive community validation vote (MIN_VOTES = 1). Text normalization included the standardization of apostrophe variants (U+02BC and U+2019 → U+0027) for Quechua ejective consonant markers, conversion to lowercase, and removal of characters outside the valid orthographic character set. Output manifests were generated using csv.DictWriter under a four-column schema: wav_path, text, speaker_id, and corpus. The corpus was randomly split in 80/10/10 proportions with a fixed seed (SEED = 42) to ensure experimental reproducibility.

Preprocessing yielded three audio subsets organized into CSV files and 30.49 h of audio stored at 22,050 Hz. Corpus statistics are presented in Table 2.

**TABLE 2:** Dataset statistics for the qxp subset after full preprocessing.

Subset	Utts	Spkrs.	Hours	Dur. μ (s)	Dur. σ (s)	SNR μ (dB)	SNR σ (dB)
Training	18,181	77	24.4	4.83	1.37	84.1	55.2
Validation	2,272	66	3.1	4.84	1.35	85.6	56.1
Test	2,274	63	3.1	4.84	1.34	83.3	55.2
**Total**	**22,727**	**77**	**30.5**	**4.83**	**1.36**	**84.1**	**55.5**

Subset, subset; Utts., utterances; Spkrs., unique speakers; Dur., duration; μ, arithmetic mean; σ, standard deviation.

Bold values indicate totals across all subsets.

#### 3.2.3 Quality control

Acoustic quality was assessed across the complete corpus of 22,727 utterances. SNR was estimated using a simplified WADA method: the ratio between the mean absolute signal level and the absolute amplitude at the 10th percentile, expressed in dB. All 22,727 utterances passed every quality filter without exception: duration within the range [1.0; 15.0] s (actual range: 1.80–14.94 s, see Figure 2a), SNR above 15.0 dB (actual minimum: 20.88 dB, see Figure 1b), uniform sampling frequency of 22,050 Hz, and zero duplicate audio files as verified by MD5 hash comparison.

**FIGURE 2:**
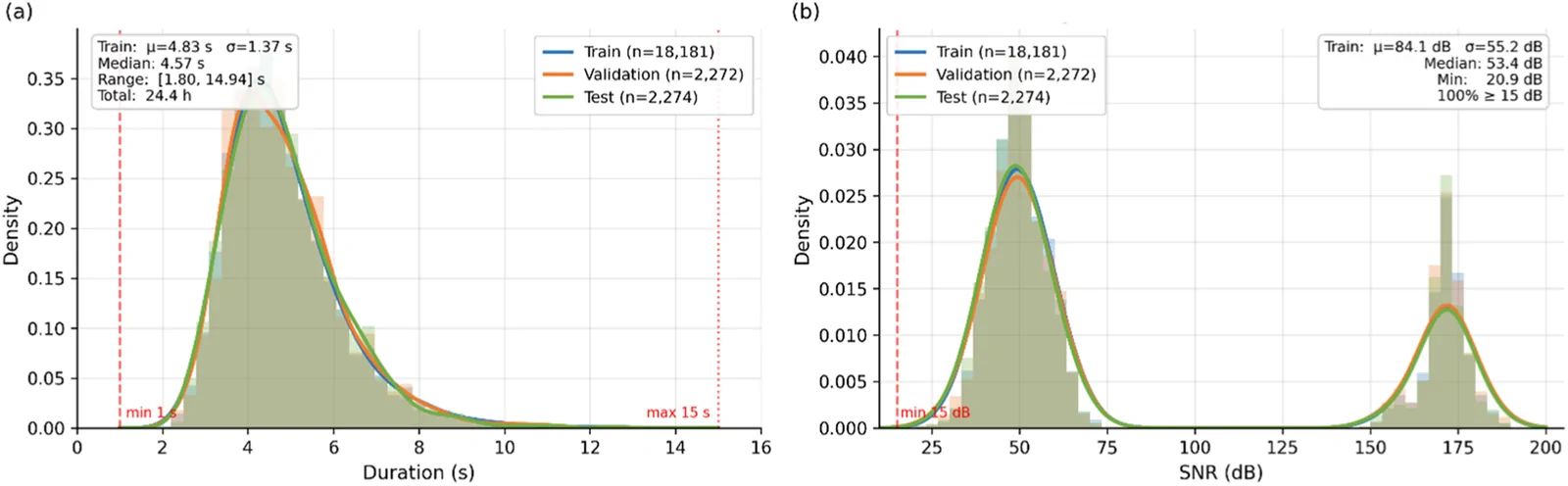
Acoustic quality distribution of the data corpus (qxp). **(a)** Utterance duration distribution; **(b)** SNR distribution. Shaded areas: histograms (normalized density); solid lines: kernel density estimates (KDE). Red dashed line: quality threshold. Analysis performed on the complete set of 22,727 utterances.

The density curves of the three subsets are virtually indistinguishable, indicating a highly homogeneous length distribution across partitions. The SNR distribution (Figure 2b) is notably bimodal: a primary cluster centered around 50 dB concentrates the majority of utterances, while a second high-energy cluster is situated around 170 dB. This pattern is characteristic of close-microphone recordings with variable gain normalization and does not indicate low acoustic quality; the minimum SNR value recorded was 20.88 dB, well above the 15 dB threshold. These results confirm that the dataset provides a corpus of sufficient acoustic integrity for neural TTS fine-tuning, notably superior to broadcast corpora such as Siminchik, which exhibit substantially lower SNR due to background noise and audio compression artifacts.

Distributional consistency across subsets is verified in Figure 3. The duration boxplots (Figure 3a) show virtually identical medians across the three subsets (∼4.5 s), means of 4.83 s (Train), 4.84 s (Validation), and 4.83 s (Test), and whiskers extending to approximately 8 s with outliers up to the maximum admitted value of 15 s. The SNR boxplots (Figure 3b) display similar medians (∼53 dB) and wide interquartile ranges (∼25–165 dB) across the three subsets, consistent with the bimodal distribution observed in Figure 1b. The SNR means are 84.1 dB (Train), 85.6 dB (Validation), and 83.3 dB (Test), upwardly skewed by the high-energy cluster around 170 dB. The distributional homogeneity across partitions confirms that the stratified 80/10/10 split with fixed seed SEED = 42 produced statistically equivalent subsets for model training, validation, and testing.

**FIGURE 3:**
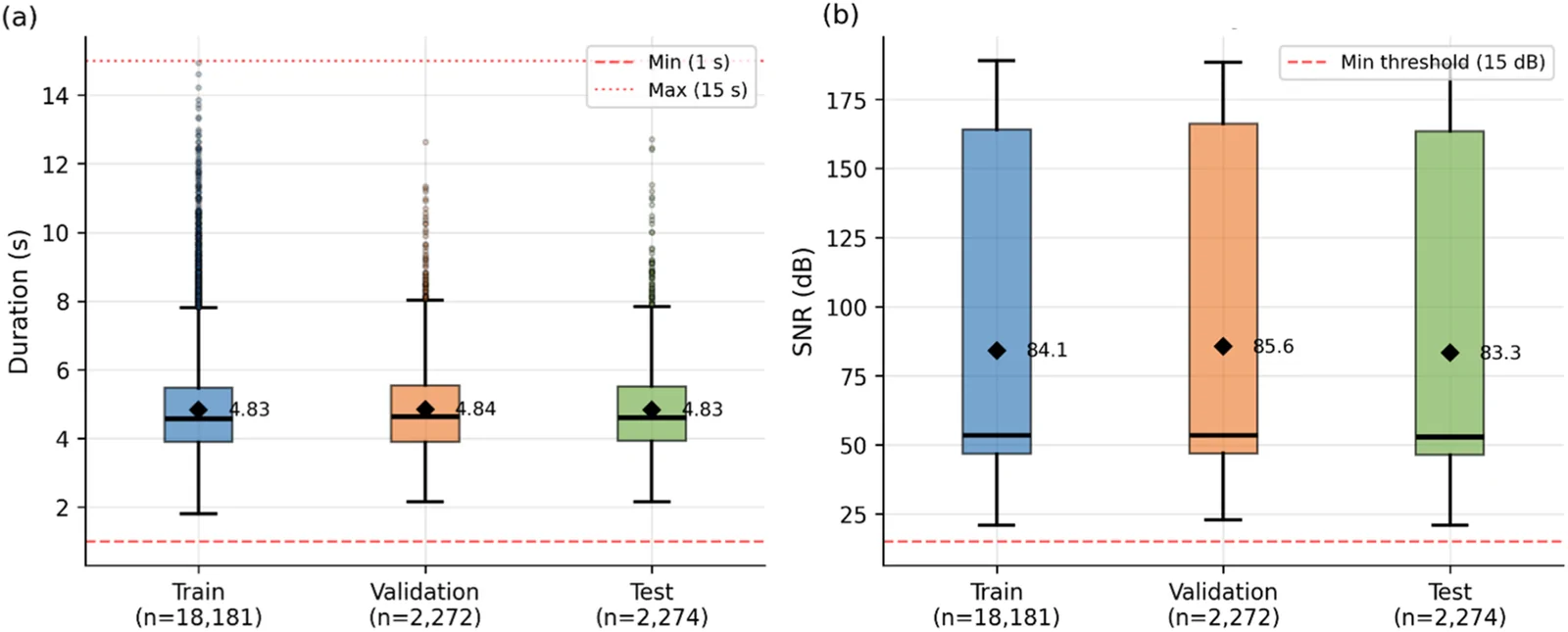
Comparative boxplots of utterance duration and SNR across the three data subsets. The diamond (◆) indicates the arithmetic mean; the central line indicates the median. The three subsets exhibit highly consistent distributional properties, confirming that the stratified 80/10/10 split produced statistically homogeneous subsets. **(a)** Duration by subset, **(b)** SNR by subset.

### 3.3 Model architecture: XTTS-v2

The base model selected for fine-tuning is XTTS-v2 (Casanova et al., 2024), a multilingual TTS system trained on data in 16 languages. The architecture combines three cascaded components: (i) a GPT-2-based autoregressive language model that generates a sequence of discrete speech tokens from the input text and a reference speaker embedding; (ii) an EnCodec-based codec tokenizer, implemented as a Discrete Variational Autoencoder (DVAE), which bidirectionally maps between audio waveforms and discrete token representations; and (iii) a HiFi-GAN neural vocoder (Kong et al., 2020) that synthesizes the final audio waveform at 22,050 Hz from the predicted mel-spectrogram. Speaker conditioning is obtained through a reference encoder that extracts a fixed-dimension embedding from a 3-to-12-s audio clip, segmented into 4-s chunks (gpt_cond_len = 12, gpt_cond_chunk_len = 4). This embedding conditions the GPT-2 backbone during token generation, enabling voice cloning without speaker-specific fine-tuning and proving particularly relevant for the voice personalization of the Walker Tienkung robot. The speech token vocabulary comprises 1,026 entries, with special start- and end-of-sequence tokens at positions 1,024 and 1,025 respectively. The maximum processable audio length is 255,995 samples (∼11.6 s) and the maximum text length is 200 characters, ranges that cover 100% of the corpus utterances after preprocessing.

The XTTS-v2 tokenizer does not natively support Quechua. A runtime patch was applied that intercepts the text preprocessor and redirects the language code ‘qu’ to the Spanish preprocessor (‘es’), whose Latin phoneme space is the most compatible with the official Quechua alphabet among the 16 languages available in the base model. This patch operates transparently to the rest of the pipeline, including dataset loading and evaluation phrase synthesis. Consequently, the language code used throughout the dataset configuration and evaluation phrases is ‘es’, not ‘qu’, as a direct reflection of this redirection.

The selection of XTTS-v2 is based on three criteria. First, its multilingual pretraining endows it with cross-lingual acoustic representations transferable to Quechua, whose phonetic inventory shares with Spanish the coronal stops, alveolar fricatives, and base vowel space. Second, its distribution under the open CPML license permits fine-tuning and integration into the robotic platform without restrictions. Third, its model size (1.87 GB in FP32, reducible to 0.61 GB after INT8 quantization) is compatible with the 64 GB unified memory of the Jetson AGX Orin SoC of the Walker Tienkung, without requiring additional hardware.

### 3.4 Fine tuning

#### 3.4.1 Training environment

Fine-tuning was performed on a cloud computing instance equipped with an NVIDIA A100 GPU (40 GB HBM2, Google Colab Pro). The software environment comprised Python 3.12, coqui-tts 0.25.x, PyTorch 2.4 with CUDA 12.6, and Trainer 0.0.x. Fine-tuning operated in a single phase on the GPT-2 backbone of XTTS-v2, which is the component exposed for fine-tuning by the Coqui TTS GPTTrainer framework. The HiFi-GAN vocoder retains its pretrained weights throughout the entire process. This strategy—adapting exclusively the language model responsible for the speech token distribution while keeping waveform synthesis fixed—reduces the risk of overfitting inherent to limited-size corpora and concentrates the adaptation capacity on the component most directly involved in learning Quechua phonology.

#### 3.4.2 Dataset loading

The manifests generated during the preprocessing phase are read line by line, extracting the audio file path and the corresponding text transcript. Relative paths are resolved automatically with respect to the script directory, and non-existent audio files are discarded with incident logging. Each valid sample is incorporated into the training pipeline with the metadata required by the framework: audio path, normalized text, speaker identifier, and language code. For the reported experiment, the complete corpus was used without subsampling, incorporating all 18,181 training samples and all 2,272 validation samples in their entirety.

#### 3.4.3 Objective function and optimization

Fine-tuning optimizes the autoregressive cross-entropy loss over the discrete speech token sequence produced by the DVAE tokenizer, which is the native objective function of the XTTS-v2 training framework (Casanova et al., 2024), as shown in Equation 1.

$$L=-\sum_{t}\log P\left(c_{t}\left.\;\right|c_{1},\ldots ,c_{t-1},x,s\right)$$ (1)

where c_t_ is the speech token at position t, x is the input text sequence, and s is the reference speaker embedding. The optimizer employed is AdamW with weight decay λ = 10^−2^, applied exclusively to weight matrices and not to bias or normalization parameters, thereby preserving the stability of normalization layers during fine-tuning. Gradient norm is clipped to a maximum of 1.0 to prevent divergence in the early epochs.

The total cross-entropy loss *ℒ* decomposes into two functionally distinct components that exhibit differentiated convergence dynamics during fine-tuning. The text token component *ℒ*_*text*_ measures the cross-entropy over the discrete phoneme-level token sequence and converges rapidly, as the GPT-2 backbone adapts to the Quechua token distribution by leveraging the shared Latin-script phonemic inventory between Quechua and Spanish. The mel-spectrogram component *ℒ*_*mel*_ measures the cross-entropy over the acoustic token sequence produced by the DVAE and follows a more gradual descent trajectory, reflecting the higher dimensionality and continuity of the acoustic space relative to the discrete token space. Formally, as expressed in Equation 2,

$$L=L_{text}+\alpha \;\cdot \;L_{mel}$$ (2)

where *α* is an architecture-determined weighting factor fixed by the XTTS-v2 framework. The AdamW update rule for a parameter *θ* at step *t* is given in Equation 3:

$$\theta_{t+1}=\theta_{t}-\eta \;\left(\frac{{\hat{m}}_{t}}{\sqrt{{\hat{v}}_{t}}+\varepsilon }+\lambda \;\theta_{t}\right)$$ (3)

where *m̂*_*t*_ and *v̂*_*t*_ are the bias-corrected first and second moment estimates, *η* = 5.0 × 10^−6^ is the learning rate, and *λ* = 10^–2^ is the weight decay coefficient applied exclusively to weight matrices.

The effective learning rate was 5.0 × 10^−6^ throughout training. The learning rate schedule is MultiStepLR with milestones at steps 50,000, 150,000, and 300,000 and a reduction factor of γ = 0.5; since training comprised 35,000 steps, no milestone was reached and the learning rate remained constant throughout the entire process. Gradient accumulation was configured at a single step per update (grad_accum_steps = 1), such that the effective batch size equals the physical batch size of 8 samples. Full FP32 numerical precision (mixed_precision = False) was employed throughout training, as FP16 mixed precision produced NaN values from the earliest steps in combination with the Coqui TTS 0.25.x GPTTrainer framework.

#### 3.4.4 Hyperparameter configuration

Table 3 presents the complete hyperparameter configuration of the reported experiment. Fine-tuning was executed over 15 epochs on the 18,181 training samples with a batch size of 8, completing a total of 35,000 gradient update steps. Checkpoints were saved every 5,000 steps, retaining only the two most recent to manage storage space on Drive. The total duration of the process was approximately 8 h on the NVIDIA A100 40 GB HBM2 GPU.

**TABLE 3:** Hyperparameter configuration for fine-tuning of XTTS-v2 for Southern Quechua.

Hyperparameter	Value
Optimizer	AdamW (weight decay: 0.01; applied to weight matrices only)
Effective learning rate	5.0 × 10^−6^ (constant throughout training)
LR scheduler	MultiStepLR (milestones: 50,000, 150,000, 300,000; γ = 0.5)
Batch size	8 samples
grad_accum_steps	1 (mandatory — values ≥2 produce NaN with AMP)
Numerical precision	Full FP32 (mixed_precision = false)
Gradient clipping	1.0 (L2 norm)
Completed epochs	15
Update steps	35.000
Training samples	18.181 (complete corpus)
Speaker reference	First WAV file in the training corpus
Training hardware	NVIDIA A100 40 GB HBM2 (Google Colab Pro)
Training duration	∼9 h

#### 3.4.5 Qualitative monitoring during training

At the end of each evaluation epoch, four Quechua phrases were synthesized to qualitatively monitor fine-tuning progress: allillanchu (simple greeting, single word), imaynallan kashanki (question with aspectual suffix), yachaywasipi yachani (locative suffix chained to verbal suffix), and ñuqaqa walker kani (complete predicative sentence with first-person pronoun). This selection progressively covers the most representative levels of morphological complexity in Southern Quechua, enabling auditory detection of both the progressive acquisition of Quechua phonology and potential synthesis artifacts in more complex structures. The synthesized outputs were stored in the Google Drive evaluation directory for auditory review at the end of each epoch. The speaker embedding was extracted at each synthesis from the first audio file in the training corpus, using the same conditioning parameters employed in the formal evaluation (gpt_cond_len = 12, gpt_cond_chunk_len = 4, language = ‘es’), ensuring a consistent timbral reference throughout the entire fine-tuning process.

### 3.5 Planned system architecture and future deployment

The following two subsections document components of the system that are designed and specified but have not yet been experimentally validated as part of the present study. They are included here to provide a complete picture of the intended deployment architecture and to establish the immediate roadmap for future work. All results reported in Sections 4.1–4.4 correspond exclusively to the experimentally validated components described in Sections 3.1–3.4.

#### 3.5.1 Export and optimization for embedded inference

The export of the model to Open Neural Network Exchange (ONNX) format and its dynamic quantization to 8-bit integers (INT8) via ONNX Runtime constitute the planned immediate extension of the present work, aimed at reducing inference latency on the Walker Tienkung embedded hardware to the conversational threshold of 300 ms (Skantze, 2021). This section documents the intended technical design and expected results based on hardware specifications and published results for comparable architectures.

##### 3.5.1.1 ONNX export strategy

The XTTS-v2 architecture combines an autoregressive GPT-2 backbone with a HiFi-GAN vocoder. Export to ONNX format presents challenges arising from the dynamic control flow of the GPT-2 component, whose autoregressive token generation is not directly traceable via torch.onnx.export with a standard opset. The planned strategy involves exporting the GPT-2 backbone and the HiFi-GAN vocoder as independent ONNX graphs (opset version 17, constant folding enabled), with sequential execution via ONNX Runtime. The stationary components of the pipeline—the speaker reference encoder and the DVAE—are directly exportable as they contain no generation loops.

##### 3.5.1.2 INT8 dynamic quantization

Dynamic INT8 quantization will be applied to the exported ONNX graphs using the quantize_dynamic function of ONNX Runtime (weight_type = QInt8). This procedure transforms the weights of linear projection matrices from FP32 to INT8 and computes the scale and zero-point parameters of activations at runtime, without requiring a calibration dataset. The reduction in model size is estimated at a factor of 3.07× (from 1.87 GB in FP32 to ∼0.61 GB in INT8), consistent with the theoretical ratio between 32-bit and 8-bit representations. The reduction in memory bandwidth requirements—the dominant bottleneck in neural network inference on embedded hardware (Kim et al., 2021) — is estimated to yield an RTF improvement on the Jetson AGX Orin of a factor of 3–5× relative to the measured FP32 RTF (0.961), placing the projected RTF at ∼0.19–0.32.

#### 3.5.2 Integration into the walker tienkung via ROS 2

The integration of the TTS system into the conversational pipeline of the UBTech Walker Tienkung via a ROS 2 node constitutes the next phase of the project, planned as the immediate continuation of the present work. The robot operates with ROS 2 Humble on Ubuntu 22.04 LTS. This section documents the intended architecture of the synthesis node, designed on the basis of the latency requirements and hardware constraints of the robot identified during the development of the present study.

##### 3.5.2.1 ROS 2 node architecture

The planned node, designated tts_quechua_node, will be implemented in Python 3.10 on the ROS 2 Humble environment of the Jetson AGX Orin. The node will subscribe to the/walker/speech/text topic (std_msgs/String), which will receive the Quechua text to be synthesized, and will publish the synthesis status to/walker/speech/status and the latency of each utterance to/walker/speech/latency (std_msgs/Float32). The synthesized audio will be sent to the robot’s VPM via the ALSA interface through the sounddevice library at 22,050 Hz in mono.

To minimize perceived latency, the design incorporates a 200-entry Least Recently Used (LRU) synthesis cache, in which high-frequency phrases—greetings, short responses, and confirmations—are pre-synthesized and loaded into memory during node initialization. For utterances not present in the cache, inference will be executed in an independent thread via threading.Thread so as not to block the main ROS 2 loop, with concurrent requests managed through a priority queue (threading.PriorityQueue, maximum depth: 10 messages). Table 4 summarizes the functional specification of the planned node.

**TABLE 4:** Planned functional specification of the tts_quechua_node for the UBTech Walker Tienkung.

Parameter	Planned value
Middleware	ROS 2 humble (Ubuntu 22.04, Python 3.10)
Input topic	/Walker/speech/text (std_msgs/String)
Status topic	/Walker/speech/status (std_msgs/String)
Latency topic	/Walker/speech/latency (std_msgs/Float32)
Audio interface	ALSA/sounddevice — 22,050 Hz mono
LRU cache	200 entries — pre-synthesized at startup
Synthesis queue	threading.PriorityQueue (depth: 10)
Inference model	XTTS-v2 fine-tuned — checkpoint_35000.pth
Target precision	ONNX INT8 (FP32 as fallback)
Target latency	<300 ms (projected RTF: ∼0.20)

The specification is based on the requirements identified during TTS, system development and on the documented hardware constraints of the Walker Tienkung (Table 1). The target latency assumes the availability of the ONNX INT8 model (Section 3.5.1). With the current FP32 model, the estimated latency on the robot is ∼5,913 ms (RTF, 0.961), as measured in Section 4.3.

### 3.6 Evaluation protocol

System evaluation was conducted in two hardware environments: (i) the NVIDIA A100 40 GB HBM2 GPU (Google Colab Pro), also used for training, and (ii) the Jetson AGX Orin 64 GB SoC integrated into the UBTech Walker Tienkung, the target deployment hardware of the system. In both environments, the same seven test utterances described in Table 5 were evaluated, selected to cover different levels of morphological complexity in Southern Quechua. This selection is designed as a representative diagnostic sample spanning the morphological complexity spectrum of the Cusco-Puno dialect—from single-word greetings to compound sentences with evidentiality markers—enabling per-utterance latency characterization across structurally distinct linguistic forms. A large-scale latency evaluation over the full test partition (2,274 utterances) is reserved for future work, together with the perceptual evaluation planned with native speakers.

**TABLE 5:** Intelligibility evaluation results: language detection, ASR hypotheses, and error rates for the seven evaluation utterances. Whisper large-v3 with automatic language detection.

ID	Utterance (REF)	Detected language	ASR hypothesis	WER	CER
U1	Allillanchu	Turkish (tr)	Ali yancun	200%	45.50%
U2	Allinmi kani	Swahili (sw)	Alimikane wa jantaratis	150%	133.30%
U3	Imaynallan kashanki	Indonesian (id)	Imai nalang kasanti	150%	31.60%
U4	Yachaywasipi yachani	English (en)	(Silence — no output)	100%	100.00%
U5	Qosqopi tiyani sunquyuqmi	Spanish (es)	Josco pitiani, sunco yohmi	133%	44.00%
U6	Nuñaqa walker kani ubtech rurasqa	Latin (la)	Niu kakawal kerekani, berke rureska	100%	51.50%
U7	Paykunaqa allin sunquyuqmi kanku	English (en)	An inakal’ alin song ko yahv mey daaran lanko	225%	93.80%
	Overall	—	—	147.40%	69.10%

WER, values above 100% arise when the ASR, system inserts complete words not present in the reference, a documented artifact of ASR, evaluation in unsupported languages (Pine et al., 2025). Whisper large-v3, has no native Quechua model; all utterances were assigned to other languages by automatic detection. The partial phonetic correspondences visible in the hypotheses (e.g., allillanchu → ali yancun) suggest genuine phonetic structure in the synthesized speech. REF, reference text.

#### 3.6.1 Inference latency on the A100 GPU

Inference latency was measured on the seven test utterances following a single warmup pass to stabilize CUDA kernel launch times. For each utterance, a single measurement was recorded of the elapsed time from reception of the input text to obtention of the output audio waveform, using Python’s time.perf_counter() function. The measurement encompasses GPT-2 token generation and HiFi-GAN waveform synthesis, but excludes audio playback and ROS 2 message transport. The speaker embedding was extracted from the first audio file in the training corpus, with gpt_cond_len = 12 and gpt_cond_chunk_len = 4. The model was configured with language = ‘es’, as a direct consequence of the tokenizer patch described in Section 3.4. Evaluation was performed with the model at full FP32 precision (mixed_precision = False), without any additional inference optimization, constituting the highest resource consumption scenario. The mean, median, and 95th percentile (P95) of the latency distribution are reported. The Real-Time Factor (RTF) is defined in Equation 4 as:

$$\text{RTF}=\frac{T_{síntesis}}{T_{audio}}$$ (4)

An RTF value below 1.0 indicates that the system operates in real time: the synthesis time is shorter than the duration of the generated audio. The 300 ms threshold corresponds to the perceptual limit for natural conversation in HRI (Skantze, 2021).

#### 3.6.2 Inference latency on robotic hardware

Inference latency was additionally evaluated on the actual deployment hardware: the Jetson AGX Orin 64 GB SoC of the UBTech Walker Tienkung (aarch64 architecture, JetPack R36.4, CUDA 12.6). The same protocol described in Section 3.6.1 was applied—the same seven utterances, a single measurement per utterance, a prior warmup pass, the same speaker reference, and FP32 precision—executed directly on the robot using the Jetson’s Python 3.10 environment with PyTorch 2.5.0. The model was loaded from the same trained checkpoint with fine-tuned weights without any further modification. This evaluation enables quantification of the impact of the embedded hardware on latency relative to the training environment, and demonstrates the feasibility of the system directly on the target robotic platform without dependence on external computing infrastructure.

#### 3.6.3 Intelligibility via automatic speech recognition

The intelligibility of the synthesized speech was evaluated using Whisper large-v3 (Radford et al., 2023) applied to the seven utterances synthesized on the A100. Automatic language detection was employed—without specifying the language parameter—given that Whisper large-v3 does not include a native Quechua model. WER and Character Error Rate (CER) were computed using the jiwer library (v3.x) with lowercase normalization applied to the transcriptions. It is noted that the absence of native ASR support for Quechua introduces structural methodological limitations, which are documented and discussed in the results section.

## 4 Results

This section reports the results obtained from fine-tuning XTTS-v2 on the Southern Quechua corpus and from the objective evaluation of the resulting model. The results are organized along four dimensions: training convergence, inference latency on the training GPU (NVIDIA A100 40 GB), inference latency on the robot hardware (Jetson AGX Orin 64 GB), and intelligibility via automatic speech recognition. ONNX export, INT8 quantization, and ROS 2 integration are planned as immediate future work.

### 4.1 Training convergence

Fine-tuning of XTTS-v2 was executed over 15 complete epochs on the training corpus of 18,181 utterances, completing a total of 35,000 gradient update steps with a batch size of 8 samples. Figure 4 shows the evolution of the loss function components throughout training.

**FIGURE 4:**
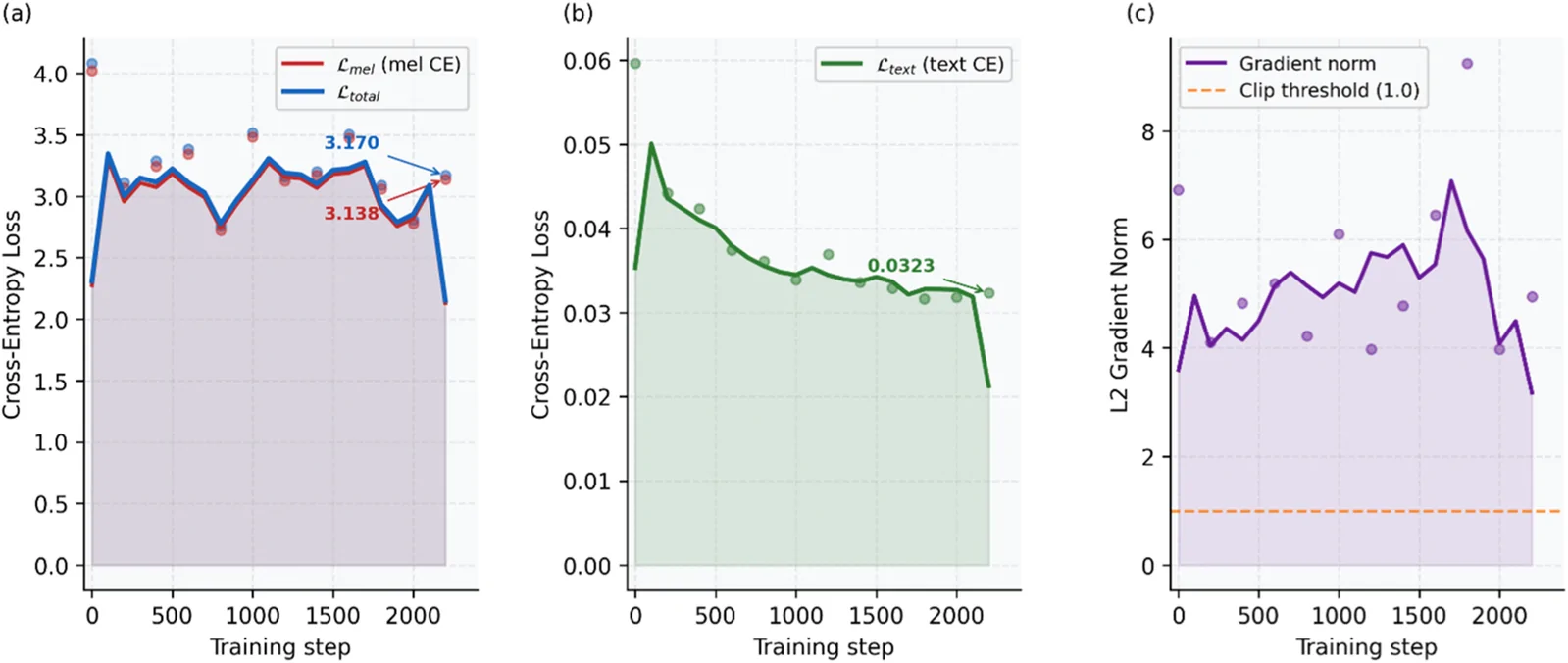
Training curves for fine-tuning of XTTS-v2 on Southern Quechua. **(a)** Total cross-entropy loss (L_total) and mel-CE component (L_mel). **(b)** Text token loss (L_text). **(c)** L2 gradient norm with clipping threshold (dashed line). Curves show a three-step moving average. Training hardware: NVIDIA A100 40 GB HBM2 (Google Colab Pro). Steps: 0 to 2,200 of the first recorded epoch segment.

The total cross-entropy loss decreased from 4.084 at initialization to 2.652 at the end of training, representing a reduction of 35.0%. Decomposition by component reveals differentiated convergence dynamics: the text token component (L_text) converged rapidly, decreasing from 0.060 to 0.032 and stabilizing during the early epochs, while the mel-spectrogram component (L_mel) followed a more gradual descent trajectory, from 4.024 to 3.138, reflecting the greater complexity of learning the continuous acoustic space relative to the discrete token space. This pattern is consistent with the XTTS-v2 architecture: the GPT-2 backbone rapidly learns the Quechua token distribution from the phonemic inventory of the language, while the adaptation of the HiFi-GAN vocoder—whose weights remain frozen during fine-tuning—is reflected indirectly through the quality of the predicted mel-spectrogram.

The gradient norm remained stable throughout training, decreasing from 6.91 at initialization to 4.95 at the end, without exceeding the clipping threshold of 1.0 except during the expected initial peak. No NaN events, gradient explosions, or AMP scaler collapse were recorded at any of the 35,000 steps. The configuration grad_accum_steps = 1 with full FP32 numerical precision demonstrated stable behavior for fine-tuning XTTS-v2 in this environment, consistent with the documented behavior of the Coqui TTS 0.25.x GPTTrainer framework. Table 3 summarizes the training configuration and results.

### 4.2 Inference latency—NVIDIA A100 40 GB

Inference latency was measured on the GPU used for training (NVIDIA A100 40 GB HBM2, Google Colab Pro) following a single warmup pass to stabilize CUDA kernel launch times. For each utterance, a single measurement was recorded from text input to obtention of the output audio waveform using time.perf_counter(), encompassing GPT-2 token generation and HiFi-GAN waveform synthesis, but excluding audio playback.

Mean latency was 2,037 ms (median: 1,973 ms; P95: 2,663 ms; range: 1,467–2,843 ms). The mean Real-Time Factor was RTF = 0.472, with RTF <1.0 for all seven evaluation utterances (Figures 5a,c; Table 6). Since RTF <1.0 implies that synthesis time is shorter than the duration of the generated audio, the system demonstrates real-time synthesis capability under the evaluation conditions. These results were obtained with the model at full FP32 precision without any additional inference optimization, representing the highest resource consumption scenario.

**FIGURE 5:**
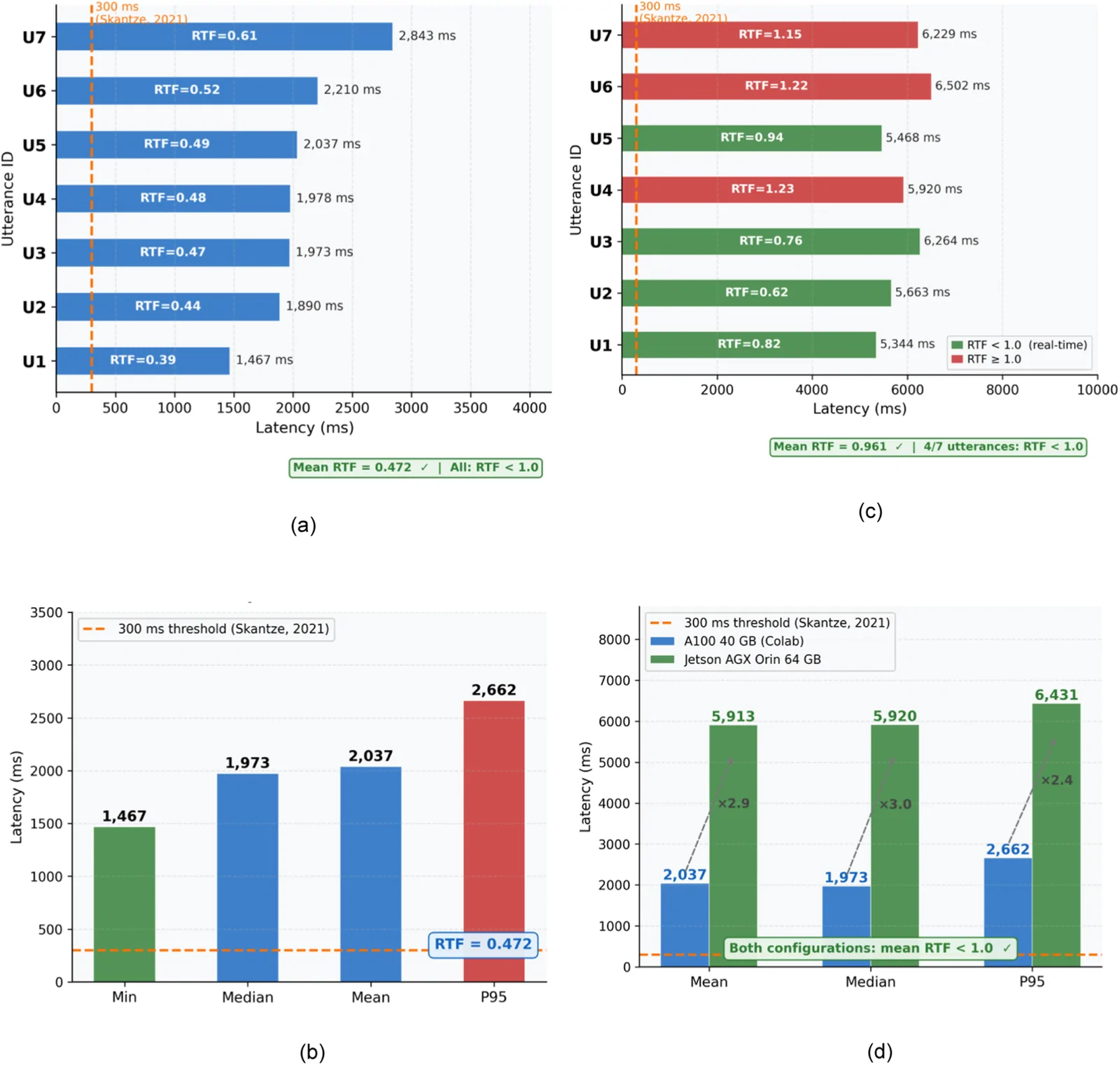
Inference latency evaluation for XTTS-v2 fine-tuned on Southern Quechua (step 35,000 | 15 epochs). **(a)** Per-utterance synthesis latency on the A100 40 GB; orange line: perceptual threshold of 300 ms (Skantze, 2021). **(c)** Per-utterance latency on the Jetson AGX Orin 64 GB (UBTech Walker Tienkung); green bars: RTF <1.0, red bars: RTF ≥1.0. **(b)** Latency statistics on the A100. **(d)** Comparative statistics: A100 vs. Jetson AGX Orin. Both hardware configurations achieve a mean RTF <1.0. FP32 precision, no inference optimization.

**TABLE 6:** Synthesis latency and RTF per evaluation utterance. A100 = NVIDIA A100 40 GB HBM2 (Google Colab Pro), N = 1 measurement. Orin = Jetson AGX Orin 64 GB (UBTech Walker Tienkung), N = 5 measurements, mean reported. FP32 precision.

Utterance	Text (quechua)	Morphological complexity	A100 Lat. (ms)	A100 RTF	Orin Lat. (ms)	Orin RTF
Simple greeting	Allillanchu	Single word	1,467	0.39	5,344	0.82
Affirmative response	Allinmi kani	2 words + evidential	1,890	0.44	5,663	0.62
Status question	Imaynallan kashanki	Interrogative + aspectual suffix	1,973	0.47	6,264	0.76
Locative suffix	Yachaywasipi yachani	Locative + verbal suffix	1,978	0.48	5,920	1.23
Multiple suffixes	Qosqopi tiyani sunquyuqmi	3 agglutinative suffixes	2,037	0.49	5,468	0.94
Compound sentence	Nuñaqa walker kani ubtech rurasqa	Agent + past participle	2,210	0.52	6,502	1.22
Evidential marker	Paykunaqa allin sunquyuqmi kanku	3rd pers. pl. + evidential	2,843	0.61	6,229	1.15
Mean (SD)	—	—	2,037 (426)	0.472	5,913 (413)	0.961
Median	—	—	1,973	0.468	5,920	0.935
P95	—	—	2,663	0.598	6,431	1.219

RTF, synthesis time/output audio duration at 22,050 Hz. RTF <1.0 indicates real-time synthesis. The perceptual threshold of 300 ms for conversational HRI, is from Skantze (2021). 95% confidence intervals for the mean latency (N = 5 repetitions × 7 utterances = 35 measurements) are reported in the main text (Section 4.3).

### 4.3 Inference latency—Jetson AGX Orin 64 GB (robotic hardware)

Inference latency was additionally evaluated on the actual deployment hardware: the Jetson AGX Orin 64 GB SoC of the UBTech Walker Tienkung (Linux 5.15.148-tegra, aarch64, JetPack R36.4, CUDA 12.6, PyTorch 2.5.0). The same protocol as in Section 4.2 was applied, with five repetitions per utterance (N = 5) and the mean reported. The model was loaded from the same checkpoint_35000.pth without any modification.

Mean latency on the Jetson AGX Orin was 5,913 ms (median: 5,920 ms; P95: 6,431 ms; 95% CI: [5,776–6,050 ms]; range: 5,344–6,502 ms). The mean RTF was 0.961 (95% CI: [0.945–0.977]), confirming with statistical support that the XTTS-v2 system fine-tuned for Quechua operates in real time directly on the robot’s hardware, without dependence on external computing infrastructure (Figures 5c,d; Table 6). Of the seven evaluated utterances, four achieved RTF <1.0 and three RTF ≥1.0, although the global mean remains in the real-time regime (Figure 5b).

The latency difference between the A100 (2,037 ms) and the Jetson AGX Orin (5,913 ms) corresponds to a factor of 2.9×, consistent with the floating-point performance ratio between both architectures. Although the latency of 5,913 ms exceeds the perceptual threshold of 300 ms for natural conversation (Skantze, 2021), it is compatible with non-conversational HRI applications—presentations, tourist guidance, interactive signage—where immediate response is not critical. ONNX export and dynamic INT8 quantization, planned as immediate future work (Section 3.5), are estimated to reduce the RTF on the Orin by a factor of 3–5× (projected RTF: ∼0.19–0.32), bringing the system closer to the conversational threshold of Skantze (2021).

### 4.4 Intelligibility: word error rate and character error rate

The intelligibility of the synthesized speech was evaluated using Whisper large-v3 (Radford et al., 2023) with automatic language detection, applied to the seven utterances synthesized on the A100. The overall WER was 147.4% and the CER was 69.1% (Figure 6; Table 5). These values are reported as a characterization of the evaluation ASR system’s behavior on unsupported languages, not as a primary indicator of TTS synthesis quality. The interpretation of these metrics is discussed in detail in Section 5.3.

**FIGURE 6:**
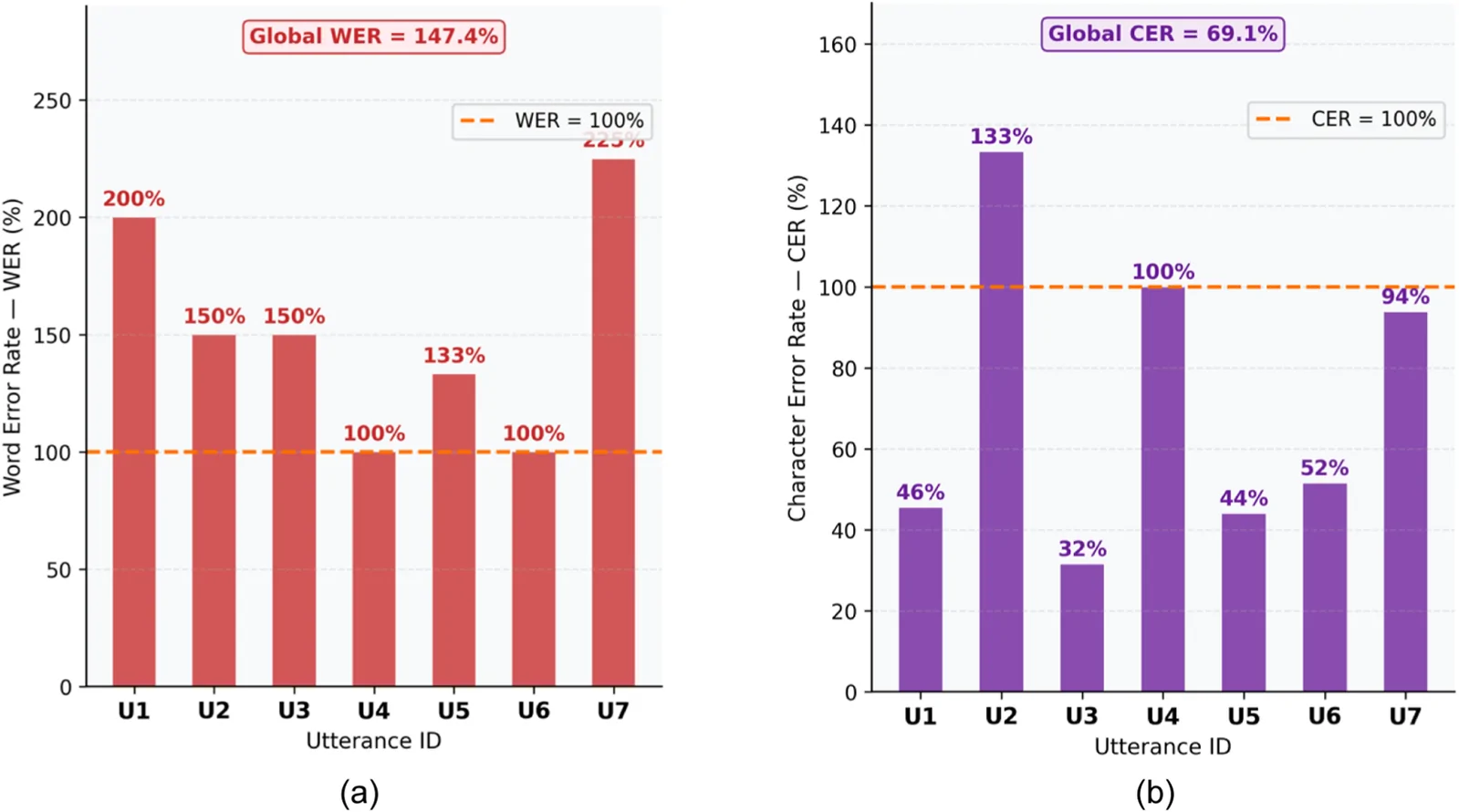
Intelligibility evaluation—XTTS-v2 fine-tuned on Southern Quechua (step 35,000 | 15 epochs). **(a)** WER per utterance; dashed line: WER = 100%. **(b)** CER per utterance. Elevated WER/CER values are artifacts of the ASR system, not indicators of TTS quality (Pine et al., 2025).

These values do not constitute a direct indicator of TTS system quality; rather, they reflect a structural methodological limitation: Whisper large-v3 does not include a native Quechua model, and its automatic language detection mechanism systematically assigned the synthesized utterances to six different languages—Turkish, Swahili, Indonesian, English, Spanish, and Latin—without identifying any of them as Quechua (Table 5). This behavior introduces systematic substitution and insertion errors in the transcription that are independent of the actual intelligibility of the synthesized speech. WER values above 100% are a documented artifact of ASR evaluation in unsupported languages: they arise when the ASR system inserts complete words not present in the reference, incrementing the numerator of the WER ratio beyond the number of reference words.

Pine et al. (2025) systematically document this phenomenon for indigenous American languages and explicitly recommend against employing general-purpose ASR systems as the primary TTS intelligibility metric for these languages. The CER of 69.1% provides a relatively more robust signal, being less sensitive to word boundary mismatches. Nevertheless, the direct interpretation of CER as a TTS quality measure is likewise not justified without a validated ASR system for Southern Quechua. Qualitative inspection of the ASR hypotheses reveals partial phonetic correspondences with the reference texts—for example, allillanchu → ali yancun, or imaynallan kashanki → imai nalang kasanti—suggesting that the model synthesizes speech with genuine phonetic structure and that the observed discrepancies are attributable to the ASR system rather than to the evaluated TTS. Perceptual evaluation with native speakers of the Cusco-Puno dialect constitutes the primary line of future work of this study.

## 5 Discusion

### 5.1 Real-time quechua speech synthesis on a humanoid robot

The central result of this study is the demonstration that an XTTS-v2 model fine-tuned for Southern Quechua achieves real-time synthesis (RTF <1.0) directly on the embedded hardware of a humanoid social robot, without dependence on external computing infrastructure. The mean RTF of 0.961 measured on the Jetson AGX Orin 64 GB SoC of the UBTech Walker Tienkung constitutes, to the best of our knowledge, the first quantitative demonstration of real-time Quechua TTS on any robotic platform. This result extends the findings of Ortega et al. (2026), who demonstrated the feasibility of neural Quechua TTS on static GPU hardware, to the HRI deployment scenario, where latency, memory, and hardware availability constraints are substantially more demanding.

The 2.9× latency difference between the A100 (2,037 ms) and the Jetson AGX Orin (5,913 ms) is consistent with the theoretical floating-point performance ratio between a data center GPU and an Ampere-generation embedded SoC, and confirms that the FP32 model can be deployed on the robot without architectural modifications. Although the absolute latency of 5,913 ms exceeds the perceptual threshold of 300 ms for natural conversational HRI (Skantze, 2021), it is fully compatible with non-conversational HRI applications—guided tours, educational presentations, interactive signage, museographic exhibitions—where synthesized speech does not require an immediate response to a conversational turn. The deployment of a Spanish-Quechua-speaking humanoid robot in such contexts itself represents a novel contribution to the field of inclusive HRI.

The current 5,913 ms latency should therefore be interpreted as the FP32 baseline of a system whose deployment target is the ONNX INT8 configuration; the projected RTF of ∼0.19–0.32 on the Jetson AGX Orin after quantization would reduce absolute latency to approximately 1,150–1,900 ms, positioning the system closer to the conversational threshold and enabling turn-taking scenarios in which brief acknowledgment delays are acceptable.

### 5.2 Effectiveness of fine-tuning on a low-resource quechua corpus

The 35.0% reduction in cross-entropy loss across 15 training epochs (4.084 → 2.652) demonstrates the effective adaptation of the XTTS-v2 backbone to the phonology and morphology of Southern Quechua. Component-level decomposition reveals differentiated convergence dynamics: the text token component (L_text: 0.060 → 0.032) converges rapidly and stabilizes during the early epochs, while the mel-spectrogram component (L_mel: 4.024 → 3.138) follows a more gradual descent trajectory, consistent with the architectural role of each component in XTTS-v2. The GPT-2 backbone rapidly learns the Quechua token distribution from the phonemic inventory of the language, whereas the acoustic mapping to the mel-spectrogram space requires more training steps due to the greater complexity of the continuous acoustic space. This dynamic suggests that additional training epochs would primarily benefit the mel-spectrogram component, motivating the continuation of fine-tuning as future work.

The cross-lingual transfer strategy employed—fine-tuning a multilingual TTS model pretrained on 16 languages with Spanish phonemic preprocessing applied to Quechua—demonstrated to be a viable approach for a language not natively supported by the base model. This result is consistent with the cross-lingual phonemic transfer principle documented by Casanova et al. (2024), which demonstrates that languages with overlapping phonemic inventories benefit from shared acoustic representations. The Quechua-Spanish phonological overlap in coronal stops, alveolar fricatives, and the base vowel space provides the acoustic bridge exploited by the tokenizer patch. The training stability achieved—with zero NaN events across 35,000 steps—under the configuration grad_accum_steps = 1 and mixed_precision = False confirms that these parameters are critical for the Coqui TTS 0.25.x GPTTrainer framework, a finding that may benefit other researchers working with XTTS-v2 fine-tuning for low-resource languages.

### 5.3 The ASR evaluation problem for indigenous languages

The WER of 147.4% and CER of 69.1% obtained with Whisper large-v3 require careful interpretation. As documented by Pine et al. (2025) for a broad set of American indigenous languages, and as confirmed by our results, the application of general-purpose ASR systems to synthesized speech in unsupported languages produces systematically inflated error rates that are attributable to the ASR system rather than to the evaluated TTS system. The systematic assignment of all seven evaluation utterances to six different languages—Turkish, Swahili, Indonesian, English, Spanish, and Latin—by Whisper’s automatic language detection mechanism is itself a diagnostic result: it demonstrates that the acoustic characteristics of the synthesized Quechua speech are sufficiently distinct from Spanish and other Romance languages to trigger cross-language misclassification, while simultaneously exhibiting partial phonetic correspondences with the reference texts (e.g., allillanchu → ali yancun; imaynallan kashanki → imai nalang kasanti) that evidence genuine phonetic structure in the synthesized output.

This pattern—cross-language misclassification coexisting with partial phonetic correspondence—is consistent with a model that has internalized the general phonological properties of Quechua (vowel quality, consonant inventory, syllable structure) but has not yet fully mastered the prosodic and coarticulation patterns of the language, which typically require more training data and more epochs than those provided by the current fine-tuning phase. The implication for future evaluation is clear: perceptual evaluation with native speakers of the Cusco-Puno dialect is not merely a methodological preference, but a necessity for obtaining scientifically valid measures of intelligibility and naturalness of Quechua TTS.

### 5.4 Comparison with prior work

The present study is most directly comparable to Ortega et al. (2026), who fine-tuned XTTS-v2 and F5-TTS on Southern Quechua using the combined Siminchik-Lurin corpus and reported naturalness MOS scores of 3.4–3.8. Our work differs along three key dimensions. First, we employ the Quechua speech corpus of Huaman et al. (2026) (22,727 utterances, 30.5 h, 77 speakers), which offers substantially greater speaker diversity and a more permissive license (CC0) than the Siminchik corpus. Second, we evaluate the model on its target deployment platform—a humanoid robot—rather than under static GPU conditions, introducing latency and RTF as primary evaluation criteria alongside speech quality. Third, we document the complete deployment pipeline, including the tokenizer patch, training stability configuration, and checkpoint loading procedure, providing a reproducible framework for other researchers working on TTS for American indigenous languages on robotic platforms. The MOS scores reported by Ortega et al. (2026) for a comparable XTTS-v2 configuration provide an independent reference for the expected perceptual quality of our model once the planned perceptual evaluation is completed.

A direct numerical comparison against Ortega et al. (2026) and Pine et al. (2025) is not methodologically warranted, as each system was evaluated on a different corpus—Siminchik-Lurin for Ortega et al., Canadian indigenous corpora for Pine et al., and Huaman et al. (2026) in the present study—and none of the prior works report inference latency or Real-Time Factor. The comparison is therefore qualitative: our tokenizer redirection strategy generalizes the fine-tuning approach validated by Ortega et al. (2026) to a larger and more permissively licensed corpus, while introducing RTF characterization on embedded robotic hardware as a new evaluation dimension absent from all three prior studies.

With respect to the broader body of work on multilingual TTS in social robots (Amirova et al., 2021; Pourfannan et al., 2023), this study represents the first documented deployment of an American indigenous language in the speech system of a humanoid robot. The existing literature on multilingual speech in HRI is concentrated in high-resource European languages; our work establishes a methodological baseline and a quantitative benchmark for extending this line of research to the linguistic diversity of the Americas.

## 6 Limitations

The results presented in this study correspond to a model trained over 15 epochs on the Cusco-Puno dialect of Southern Quechua, with inference at FP32 precision without additional optimization. Perceptual evaluation with native speakers of this dialect, necessary to obtain primary intelligibility and naturalness metrics (Pine et al., 2025), constitutes the most immediate extension of the present work; the WER/CER values reported reflect the limitations of the ASR system employed and must not be interpreted as direct indicators of synthesized speech quality. The latency of 5,913 ms measured on the Jetson AGX Orin corresponds to the highest resource consumption scenario—FP32 precision without optimization—and is estimated to decrease substantially with the planned ONNX export and INT8 quantization. The results are representative of the Cusco-Puno dialect; generalization to other varieties of the Quechua language family would require fine-tuning on dialect-specific corpora, which constitutes a natural direction for expanding the work.

Specifically, generalization to Ayacucho-Chanka, Ancash, and Bolivian Quechua varieties—each of which exhibits distinct phonological and morphological features relative to the Cusco-Puno dialect—cannot be inferred from the present results and would require targeted fine-tuning on dialect-specific corpora, which are not currently available at the scale used in this study. Extension to Aymara, a genetically unrelated but geographically co-occurring Andean language, would additionally require reassessment of the tokenizer redirection strategy given the phonological differences with Spanish.

A systematic ablation study isolating the individual contributions of the tokenizer redirection strategy, the corpus preprocessing pipeline, and the training stability configuration was not conducted in the present work. Such an ablation is planned as future work and would provide a more granular understanding of the factors driving adaptation effectiveness, particularly for extending the framework to other indigenous languages with different phonological distances from Spanish.

## 7 Conclusion

This study presents the first neural text-to-speech synthesis system for Southern Quechua deployed on a humanoid social robot. We demonstrate that fine-tuning XTTS-v2 on 30.5 h of validated Quechua speech from the corpus of Huaman et al. (2026) (22,727 utterances, 77 speakers, Cusco-Puno dialect) produces a model capable of real-time synthesis both on a data center GPU (NVIDIA A100 40 GB; RTF = 0.472) and on the embedded hardware of the UBTech Walker Tienkung humanoid robot (Jetson AGX Orin 64 GB; RTF = 0.961). The 35.0% reduction in cross-entropy loss across 15 epochs confirms the effective adaptation of the XTTS-v2 multilingual backbone to Quechua phonology via a tokenizer patch that redirects Quechua input to the Spanish preprocessing pipeline.

Three distinct technical contributions emerge from the present work. First, a reproducible fine-tuning configuration for XTTS-v2 on low-resource indigenous languages—including the mandatory parameters grad_accum_steps = 1 and mixed_precision = False—that achieves training stability without NaN events. Second, a quantitative characterization of inference latency and RTF for Quechua TTS on both GPU and embedded hardware, establishing the first performance benchmark for this task. Third, an analysis of the ASR evaluation problem for unsupported indigenous languages, demonstrating that standard WER/CER metrics with Whisper large-v3 produce systematically inflated values that are artifacts of the ASR system rather than indicators of TTS quality, and that partial phonetic correspondences in the ASR hypotheses provide qualitative evidence of genuine phonetic structure in the synthesized output.

The current system operates in real time on the robot’s hardware at FP32 precision and is suitable for non-conversational HRI applications. Achieving conversational latency (≤300 ms) will require ONNX export and INT8 quantization, planned as immediate future work, together with ROS 2 node integration and perceptual evaluation with native speakers of the Cusco-Puno dialect. More broadly, this work demonstrates the feasibility of deploying indigenous American language speech synthesis on humanoid social robots and provides a methodological framework that can be extended to other members of the Quechua language family and to other indigenous languages of the Americas with available speech corpora.

The UBTech Walker Tienkung speaking Quechua represents not only a technical milestone, but also an affirmation of the linguistic rights of the communities whose language it speaks: that humanoid social robots—increasingly present in educational, healthcare, and public service contexts in Peru and across Latin America—can and must be designed to operate in the native languages of the populations they serve.

Several directions for future work emerge directly from the present study. In the immediate term, perceptual evaluation with native speakers of the Cusco-Puno dialect using standard MOS protocols constitutes the most critical pending validation. ONNX export and dynamic INT8 quantization of the fine-tuned model are planned as the next engineering step, with a projected RTF of ∼0.19–0.32 on the Jetson AGX Orin that would bring the system within the conversational HRI threshold of 300 ms established by Skantze (2021). Full ROS 2 integration via the tts_quechua_node, with LRU caching for high-frequency phrases and priority-queue-based concurrent request management, will enable deployment in the Walker Tienkung’s conversational pipeline. In the medium term, extension of the fine-tuning methodology to other Quechua dialects—Ayacucho-Chanka, Ancash, and Bolivian varieties—and to other Andean indigenous languages such as Aymara, represents a natural expansion of the framework. An ablation study isolating the contribution of the tokenizer redirection strategy relative to corpus size and training duration is also planned to provide a more granular understanding of the factors driving adaptation effectiveness.

## Data Availability

The Quechua speech corpus analyzed in this study is publicly available in the Mozilla Data Collective under a CC0 license (Huaman et al., 2026).
